# Conventional Treatments Cannot Improve Outcomes of Early-Stage Primary Breast Marginal Zone Lymphoma

**DOI:** 10.3389/fonc.2020.609512

**Published:** 2021-04-13

**Authors:** Hailing Liu, Jing Zhang, Lin Quan, Lei Cao, Yi Miao, Xiaoli Zhao, Haorui Shen, Li Wang, Wei Xu, Jianyong Li, Lei Fan

**Affiliations:** ^1^ Department of Hematology, Jiangsu Province Hospital, The First Affiliated Hospital of Nanjing Medical University, Collaborative Innovation Center for Cancer Personalized Medicine, Nanjing, China; ^2^ Department of Respiratory, Nanjing Chest Hospital, Chest Hospital District, Nanjing Brain hospital affiliated to Nanjing Medical University, Nanjing, China

**Keywords:** primary breast marginal zone lymphoma, surveillance, epidemiology, and end results (SEER) database, treatments, outcomes, overall survival, disease-specific survival

## Abstract

**Introduction:**

Primary breast marginal zone lymphoma (PBMZL) is a rare occurrence and less is known about its characteristics, treatments, and outcomes.

**Methods:**

We retrospectively reviewed 370 cases of early-stage PBMZL from the Surveillance, Epidemiology, and End Results database. Statistical analyses were performed to describe clinical features, determine prognostic factors, and compare different therapeutic strategies.

**Results:**

At a median follow-up of 68.5 months, the 5-year overall survival (OS) and disease-specific survival (DSS) rate were 81.2 and 95.4%, respectively. We divided the cohort into four treatment groups and compared their characteristics and survival: radiotherapy (RT) ± surgery (Sx) (n = 142, 38.4%), Sx alone (n = 71, 19.2%), any chemotherapy (CT) (n = 63, 17.0%), and none of the above (n = 94, 25.4%). Age of onset and laterality of lesions tended to relate to the choice of different treatments. Multivariate Cox analysis showed that advanced age (>60 years), concomitant tumor, and any CT (*vs* RT ± Sx) predicted poorer OS, while for DSS, there was no meaningful indicator (P > 0.05). Patients aged >60 years or treated with any CT seemed to have shorter DSS, but the difference only approached statistical significance. Then we applied a propensity score-matched analysis to demonstrate that neither RT- nor Sx-containing therapy could bring a better OS or DSS. The competing risk model suggested that CT was the only contributor to higher PBMZL-specific mortality.

**Conclusion:**

Our results show an indolent behavior of early-stage PBMZL with long-term survival. Conventional oncological treatments fail to bring survival benefits; especially CT is detrimental to survival, suggesting that observation may be advisable in the management of early-stage PBMZL, and further research on novel targeted agents is warranted for patients in need.

## Introduction

Extranodal marginal zone lymphoma (EMZL), a unique type distinguished from other nodal B-cell lymphomas, constitutes 7 to 8% of newly diagnosed lymphomas (1). It can arise in various organ sites, such as orbit, salivary gland, thyroid, lung, breast, stomach, skin, and even the dura, among which the gastric involvement is the most frequent and best-studied. Primary breast lymphoma (PBL) represents approximately 1% of non-Hodgkin lymphomas and 0.5% of all breast malignancies, thus making primary breast marginal zone lymphoma (PBMZL) a rare occurrence despite being the second most common histologic subtype of PBL (2–4). An increase of incidence in PBMZL has been observed from 2000 to 2013 with an annual percentage change of 2.3%, which is of unclear etiology, partly due to the improved recognition of this disease by pathologists (2). Elderly female patients are predominantly affected, with a median age at diagnosis of 68 years, which is owing to an estrogen-related mechanism probably (5).

Early-stage diseases and optimistic outcomes tend to be presented in EMZL, with a 5-year overall survival (OS) rate of nearly 90% and a 10-year OS rate of 70 to 80% (6–8). Comparable good prognosis has been found in PBMZL, with the 5-year progression-free survival and OS 56 and 92% respectively (9). Concerning treatments, surgery (Sx), radiotherapy (RT), and chemotherapy (CT) are mostly recommended and adopted with expert experience. Nevertheless, clinical evidence is very limited. Over the past decade, an increase has been found in the proportion of PBL patients receiving RT alone and no Sx or RT, meanwhile a decrease in Sx alone and Sx plus RT (2). Antibiotic therapies have taken their place in other EMZL owing to an etiologic link between microorganisms and these lymphomas, such as gastric (Helicobacter pylori) and ocular adnexal (Clamydia psittaci) EMZL (10, 11). However, such correlations have not been identified regarding PBMZL. In a recent study, immunochemotherapy showed superior efficacy in EMZL (12). However, concerning PBMZL, information is scarce and unrepresentative for most of the previous publications were small-scale, and the cases of PBMZL accounted for a minority. The principles of the current treatment of PBMZL refer to general early-stage EMZL, mainly local treatment, including RT and Sx, without taking into account the particularity of PBMZL.

As a result, we conducted a comprehensive population-based study, reviewing the clinical characteristics and long-term outcomes of a cohort of early-stage PBMZL from the Surveillance, Epidemiology, and End Results (SEER) database. The study also aims to discover potential prognostic factors and an optimal treatment modality for early-stage PBMZL.

## Materials and Methods

### Patients

Data on patients with PBMZL were extracted from the SEER database, which includes 18 registries that cover 30% of the United States population and collects demographic, clinical, and survival information on cancers. We identified 507 patients with early-stage PBMZL using inclusion criteria of the International Classification of Diseases for Oncology, Third Edition histology code 9699, primary site codes C50.0 through C50.6, C50.8, C50.9, and an Ann Arbor stage I or II. Individuals without complete variables (n = 130) and histological diagnosis (n = 7) were excluded. Ultimately, we incorporated 370 cases diagnosed between 1998 and 2015 into our study. The elements collected for each case were the age of onset, sex, race, primary site, laterality, Ann Arbor stage, concomitant tumor, calendar year of diagnosis, treatment modalities, survival time, survival status, and cause of death.

### Statistical Analysis

Study endpoints were OS (calculated from the time of initial diagnosis to death or the last follow-up without events) and disease-specific survival (DSS, defined as the interval from diagnosis until death due to PBMZL or the last follow-up without events). We generated survival curves *via* the Kaplan–Meier (KM) method and log-rank test and analyzed predictors of survival using the univariate and multivariate Cox regression analyses. A propensity score-matched (PSM) analysis, using the 1:1 nearest neighbor technique with a small caliper of 0.03 to ensure better balance, was performed to re-evaluate the impact of different treatments on survival in matched couples. To further evaluate the factors that contributed to PBMZL- and other cause-specific mortality, a competing risk analysis was used. Categorical variables were compared using Pearson’s χ2 or Fisher’s exact test. We conducted these analyses with STATA MP, version 13.0 (StataCorp LP, College Station, TX). Two-tailed P values <0.05 were considered statistically significant.

## Results

### Patient Characteristics and Outcomes

A total of 370 PBMZL patients with stage I (n = 323, 87.3%) or stage II (n = 47, 12.7%) disease entered the final analyses. The median age of onset is 69 years (range 24 to 93 years). There is a strong female predominance with a female:male ratio of approximately 23.7:1 (355 *vs* 15). 97.3% of cases (n = 360) were unilateral breast disease, presentation of which was almost balanced between the left and right breasts (176 *vs* 184). In regard to the specified primary sites, overlapping lesion (n = 81, 21.9%) was the most common, followed by local lesion including upper-outer quadrant (n = 73, 19.7%), upper-inner quadrant (n = 40, 10.8%), lower-inner quadrant (n = 26, 7.0%), lower-outer quadrant (n = 14, 3.8%), central portion (n = 13, 3.5%), axillary tail (n = 7, 1.9%), and nipple (n = 2, 0.5%). The primary sites of the remaining 114 cases were not specified. 35.9% of cases (n = 133) had concomitant tumor in addition to PBMZL. The therapeutic regimens, in decreasing frequency of occurrence, were as follows: no RT, Sx, or CT (n = 94, 25.4%), RT alone (n = 86, 23.2%), Sx alone (n = 71, 19.2%), Sx + RT (n = 56, 15.1%), CT alone (n = 25, 6.8%), Sx + CT (n = 17, 4.6%), RT + CT (n = 12, 3.2%), and Sx + RT + CT (n = 9, 2.5%). Taken together, RT-, Sx-, and CT-containing treatment strategies, were used in 44.0% (n = 163), 41.3% (n = 153) and 17.0% (n = 63) of patients, respectively. We further divided the 370 cases into four groups: RT ± Sx (n = 142, 38.4%), Sx alone (n = 71, 19.2%), any CT (n = 63, 17.0%), and none of the above (n = 94, 25.4%). When comparing the characteristics among the four groups, it turned out that the choice of treatment modality is related to age of onset and laterality of lesions (**Table 1**).

**Table 1:** Patient and tumor characteristics of primary breast marginal zone lymphoma among different treatments groups.

Characteristic		Total	RT ± Sx	Sx alone	Any CT	None of the above	P value
		No. %	No. %	No. %	No. %	No. %	
Distribution		370 (100)	142 (38.4)	71 (19.2)	63 (17.0)	94 (25.4)	
Age	≤60 years	114 (30.81)	57 (40.14)	16 (22.54)	29 (46.03)	12 (12.77)	**<0.001**
	>60 years	256 (69.19)	85 (59.86)	55 (77.46)	34 (53.97)	82 (87.23)
Sex	Female	355 (95.95)	136 (95.77)	69 (97.18)	59 (93.65)	91 (96.81)	0.774
	Male	15 (4.05)	6 (4.23)	2 (2.82)	4 (6.35)	3 (3.19)
Race	White	314 (84.87)	112 (78.87)	65 (91.55)	54 (85.71)	83 (88.30)	0.062
	Other	56 (15.13)	30 (21.13)	6 (8.45)	9 (14.29)	11 (11.70)
Ann Arbor stage	I	323 (87.30)	128 (90.14)	65 (91.55)	51 (80.95)	79 (84.04)	0.146
	II	47 (12.70)	14 (9.86)	6 (8.45)	12 (19.05)	15 (15.96)
Calendar year of diagnosis	1998–2005	124 (33.51)	45 (31.69)	30 (42.25)	21 (33.33)	28 (29.79)	0.357
	2006–2015	246 (66.49)	97 (68.31)	41 (57.75)	42 (66.67)	66 (70.21)
Primary site*	Local lesion	175(68.36)	72(68.57)	33(63.46)	32(76.19)	38(66.67)	0.605
	Overlapping lesion	81(31.64)	33(31.43)	19(36.54)	10(23.81)	19(33.33)
Laterality	Bilateral	10 (2.70)	1 (0.70)	1 (1.41)	5 (7.94)	3 (3.19)	**0.030**
	Unilateral	360 (97.30)	141 (99.30)	70 (98.59)	58 (92.06)	91 (96.81)
	Left	176 (47.60)	82 (57.75)	29 (40.84)	23 (36.51)	42 (44.68)	
	Right	184 (49.70)	59 (41.55)	41 (57.75)	35 (55.55)	49 (52.13)
Concomitant tumor	No	237 (64.05)	97 (68.31)	44 (61.97)	42 (66.67)	54 (57.45)	0.359
	Yes	133 (35.95)	45 (31.69)	27 (38.03)	21 (33.33)	40 (42.55)

*The primary sites of 114 cases were not specified. CT, chemotherapy; RT, radiotherapy; Sx, surgery. Bold values mean statistically significant.

At a median follow-up of 68.5 months, the 5-year OS and DSS rate were (81.2 ± 2.2) % (95% confidence interval [CI] 76.4–85.2%) and (95.4 ± 1.4%)% (95% CI 91.9–97.5%), respectively. The 10-year OS and DSS rate was (60.7 ± 3.3%) (95% CI 53.9–66.9%) and (90.2 ± 2.5%) (95% CI 83.8–94.1%), respectively. The median OS was (156 ± 7.5) months (95% CI 136–170). The median DSS was not reached. A total of 112 (31.8%) women and five (33.3%) men died, with an all-cause mortality rate of 31.6%. PBMZL- and other cause-specific mortality rates were 5.6% (n = 21) and 25.9% (n = 96), respectively. For the latter, the most frequent cause was angiocardiopathy (n = 21, 5.7%), followed by non-PBMZL hematological malignancies (n = 18, 4.9%), respiratory disorders (n = 17, 4.6%), and cerebrovascular diseases (n = 8, 2.2%).

### Relationship Between Survival and Clinical Features

**Figure 1:**
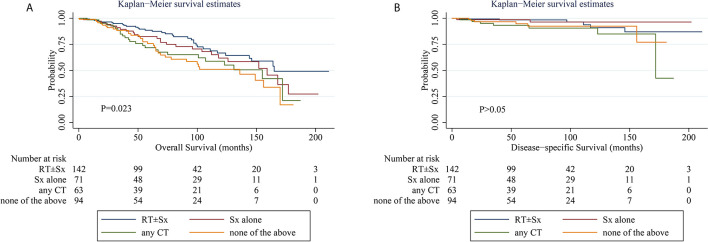
Kaplan–Meier curves of overall survival **(A)** and disease-specific survival **(B)** stratified by treatments. CT, chemotherapy; RT, radiotherapy; Sx, surgery. Number at risk indicates the number of patients with early-stage PBMZL at risk at the indicated time interval. Differences were considered statistically significant at P < 0.05.

When comparing the KM survival curve for OS among the four treatment groups, the P value among groups is 0.023, and the RT ± Sx group had the best OS graphically (**Figure 1A**). In the univariate Cox analysis, it was further demonstrated that patients treated with any CT (hazard ratio [HR] = 1.793, 95% CI 1.065–3.017, P = 0.028) and without RT, Sx, or CT (HR = 2.019, 95% CI 1.248–3.265, P = 0.004) had worse OS compared to those with RT ± Sx. Besides, age >60 years (HR = 7.297, 95% CI 3.798–14.019, P < 0.001) and concomitant tumor (HR = 1.969, 95% CI 1.369–2.833, P < 0.001) were also correlated with significantly poorer OS. In the multivariate analysis, age>60 years (HR = 6.819, 95% CI 3.518–13.217, P < 0.001), concomitant tumor (HR = 1.661, 95% CI 1.152–2.394, P = 0.007), and CT (*vs* RT ± Sx, HR = 1.733, 95% CI 1.028–2.921, P = 0.039) were independent adverse predictors for OS (**Table 2**). About the KM curves for DSS, there was no significant difference among the four groups (**Figure 1B**). Both in univariate and multivariate analysis, patients aged >60 years or treated with CT seemed to have shorter DSS, but all P values were just near statistical significance (**Table 2**). Considering that age of onset and laterality of lesions have shown significant correlations with interventions in **Table 1**, we further evaluated the impact of conventional treatments on survival in subgroups of different ages. Neither OS nor DSS differed significantly among the four treatment groups, whether in young (age ≤60 years) or old (age >60 years) cohorts (**Supplementary Figure 1**) cohorts. Given the bilateral lesion was a rare event in our cohort (n = 10, 2.7%) and had no prognostic implication, subgroup analysis was not conducted. Notably, half of the 10 bilateral PBMZL underwent CT-containing treatments.

**Table 2:** Univariate and multivariate Cox regression analyses of overall survival and disease-specific survival.

		Overall Survival				Disease-specific Survival			
Parameters		Univariate analysis		Multivariate analysis		Univariate analysis		Multivariate analysis	
		HR (95% CI)	P value	HR (95% CI)	P value	HR (95% CI)	P value	HR (95% CI)	P value
Sex (female)		1.037 (0.423–2.542)	0.937	–	–	0.934 (0.125–6.972)	0.947	–	–
Age (>60 years)		7.297 (3.798–14.019)	**<0.001**	6.819 (3.518–13.217)	**<0.001**	2.849 (0.943–8.604)	**0.063**	3.024 (0.984–9.288)	**0.053**
Ann Arbor Stage (II)		1.344 (0.803–2.250)	0.261	–	–	1.787 (0.599–5.329)	0.298	–	–
Race (White)		1.059 (0.605–1.852)	0.841	–	–	0.629 (0.211–1.875)	0.405	–	–
Primary site (overlapping lesion)		1.238 (0.780–1.964)	0.366	–	–	0.677 (0.188–2.433)	0.550	–	–
Laterality (bilateral)		0.677 (0.167–2.744)	0.585	–	–	1.990 (0.265–14.930)	0.503	–	–
Concomitant tumor (yes)		1.969 (1.369–2.833)	**<0.001**	1.661 (1.152–2.394)	**0.007**	0.754 (0.292–1.949)	0.560	–	–
Calendar year of diagnosis (≤2005)		1.256 (0.837–1.884)	0.272	–	–	1.920 (0.710–5.190)	0.198	–	–
Therapy	RT ± Sx	Reference		Reference		Reference		Reference	
	Sx alone	1.403 (0.834–2.360)	0.202	1.014 (0.600–1.713)	0.959	0.633 (0.128–3.138)	0.576	0.513 (0.103–2.567)	0.417
	Any CT	1.793 (1.065–3.017)	**0.028**	1.733 (1.028–2.921)	**0.039**	2.781 (0.931–8.306)	**0.067**	2.705 (0.907–8.065)	**0.074**
	None of the above	2.019 (1.248–3.265)	**0.004**	1.316 (0.809–2.140)	0.268	1.946 (0.623–6.080)	0.252	1.479 (0.984–9.288)	0.507

CI, confidence interval; CT, chemotherapy; HR, hazard ratio; RT, radiotherapy; Sx, surgery. Bold values mean statistically significant.

### Propensity Score-Matched Analysis

**Figure 2:**
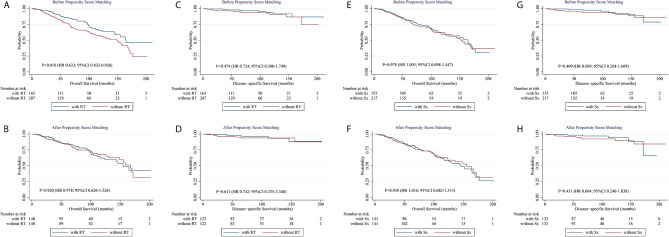
Kaplan-Meier survival curves stratified by treatments before and after propensity score matching. **(A, B)** Kaplan-Meier curves of overall survival stratified by RT-containing treatments before **(A)** and after **(B)** propensity score matching. **(C, D)** Kaplan-Meier curves of disease-specific survival stratified by RT-containing treatments before **(C)** and after **(D)** propensity score matching. **(E, F)** Kaplan-Meier curves of overall survival stratified by Sx-containing treatments before **(E)** and after **(F)** propensity score matching. **(G, H)** Kaplan-Meier curves of disease-specific survival stratified by Sx-containing treatments before **(G)** and after **(H)** propensity score matching. CI, confidence interval; HR, hazard ratio; RT, radiotherapy; Sx, surgery.

To further corroborate the influence of different strategies on prognosis, a PSM analysis was carried out (**Figure 2**). Considering the imbalance in characteristics between patients treated with and without a specific modality, we applied a 1:1 PSM ratio with a small caliper of 0.03 for matching the potential cofounders including the age of onset, sex, race, laterality, Ann Arbor stage, concomitant tumor, calendar year of diagnosis and other treatment modalities not analyzed. The distribution of covariates was adequately balanced and evenly distributed in the matched groups (**Supplementary Table 1** and **Supplementary Table 2**). Before matching, patients receiving RT-containing treatments had a superior OS than those who did not. Nevertheless, after matching, RT-containing treatment failed to show its efficacy in OS. As for DSS, no significant difference was found between the above two groups, whether unmatched or matched. Neither OS nor DSS differed significantly between patients treated with Sx-containing therapy and those who did not, before or after matching. These results suggest that neither RT nor Sx can bring survival benefits for early-stage PBMZL. PSM for CT was not achieved due to the limited number of cases, but the any CT group had a poorer OS than the RT ± Sx group, which demonstrated that CT-containing therapy fails to bring a better OS as well.

### Competing Risk Analysis

Considering the unclear impact of CT on DSS in the Cox regression, we performed a competing risk analysis to explore the risk factors that account for PBMZL- and other cause-specific mortality. According to the results above, age of onset, concomitant tumor, and CT were brought into the analysis. As shown in **Table 3**, model 1, taking into account other cause-specific mortality as the competing risk, illustrated that CT-containing treatment contributed to death from early-stage PBMZL (subdistribution hazard ratio [SHR] = 2.618, 95% CI 1.098–6.240, P = 0.030). Model 2, taking into account PBMZL-specific mortality as the competing risk, revealed that age of onset (SHR = 8.930, 95% CI 3.940–20.238, P < 0.001) and concomitant tumor (SHR = 2.109, 95% CI 1.406–3.163, P < 0.001) correlated to death due to other causes. The P values of the above models were 0.034 and <0.001, respectively. In conclusion, although age and concomitant tumor are independent prognostic factors for OS of early-stage PBMZL, they do not independently predict the survival of PBMZL itself. CT is unfavorable to the survival of early-stage PBMZL.

**Table 3:** Two competing risk models for early-stage PBMZL.

Model	Failure event	Competing event	Parameters	SHR (95% CI)	P value
1	PBMZL- specific mortality	Other cause-specific mortality	Age > 60 years	2.406 (0.794–7.297)	0.121
			Concomitant tumor	0.554 (0.206–1.494)	0.244
			Any CT	2.618 (1.098–6.240)	**0.030**
2	Other cause-specific mortality	PBMZL- specific mortality	Age > 60 years	8.930 (3.940–20.238)	**<0.001**
			Concomitant tumor	2.109 (1.406–3.163)	**<0.001**
			Any CT	1.323 (0.743–2.354)	0.342

CI, confidence interval; CT, chemotherapy; PBMZL, primary breast marginal zone lymphoma; SHR subdistribution hazard ratio.

## Discussion

To the best of our knowledge, this work has been the largest contemporary series investigating early-stage PBMZL. Patient and tumor characteristics of our cohort are similar to those reported previously (2, 9, 13). Consistent with the increasing incidence mentioned above, patients diagnosed from 2006 to 2015 were twice the population from 1998 to 2005. The good outcome confirmed the indolent nature of this lymphoma, as a 5-year OS rate of 81.2% and 10-year OS rate of 60.7%, along with a 5- and 10-year DSS rate of higher than 90%. In the survival analysis, we applied the PSM analysis to minimize the chance for error and bias, finding out that neither RT nor Sx can bring better survival. Moreover, considering the median age of onset was 69 years, there was a high risk of death from other causes. Under these circumstances, a competing risk analysis was performed and the three significant variables in the multivariate analysis were included. Noticeably, CT, rather than age and concomitant tumor, is shown to associate with higher PBMZL-specific mortality.

Due to the small number of publications reviewing PBMZL, no definite guidelines for treatment have emerged. RT, offering effective local control, appeared to be the preferred remedy for most patients (9, 14–16). Nevertheless, our study reveals that the addition of RT failed to bring a better OS or DSS. Similarly, in a large cohort of early-stage EMZL, the association of OS with RT was not significant except for cutaneous and ocular sites (17). Moreover, an inferior outcome and higher risk of recurrences were more common for other organ sites, including the breast, than gastric and thyroid EMZL among those who underwent RT (14–16). Part of the answer may be that breast radiation can cause a broad spectrum of complications including lung injury, myocardial fibrosis, and secondary malignancies (18–20). These results indicate that RT, though a suitable option for localized diseases, is met with skepticism for treating early-stage PBMZL. The role of Sx in the treatment of EMZL has declined since it has not been shown to achieve superior outcomes than less invasive interventions, supported by our study and those describing other organ sites (21, 22). In a study abstracting 465 patients’ information with PBL from 92 publications, mastectomy provided no survival benefit or protection from recurrence (23). Sx is better as an approach to obtain histopathology when faced with a diagnostic dilemma, rather than a curative strategy. A previous study of 180 nongastric EMZL (2% PBMZL) from the International Extranodal Lymphoma Study Group (IELSG) demonstrated that CT, with or without anthracycline, had no significant effect on OS, cause-specific survival, and progression-free survival, neither in localized nor in advanced-stage disease (24). Furthermore, patients with EMZL, both localized and disseminated disease, receiving CT had the worst prognosis (25). Our findings confirmed this result and further pointed out that CT is not only an independent adverse prognostic factor for OS but also a contributor to specific death from early-stage PBMZL. It is noteworthy that in our research, there is no clear advantage for any type of therapy, including RT, Sx, and CT, alone or in combination, consistent with the study from IELSG (24). The clinical activity of immunotherapy in EMZL has been proved in different extranodal organs (26–28). In the recent IELSG-19 study, immunochemotherapy (rituximab in combination with chlorambucil) showed superior efficacy compared to immunotherapy or CT alone in EMZL. However, improvements in short term control did not translate into longer OS (12). It is noteworthy that these studies tended to include cases with the gastric site, advanced stage, and relapse disease, early-stage PBMZL rarely seen. Regarding the group undergoing none of the conventional treatments in our cohort, it was not homogenous. We infer that observation and immunotherapy were the most likely policies, considering almost half of these patients were diagnosed before 2006 and the remaining were diagnosed in the post-rituximab era. Both univariate and multivariate analysis indicated that the calendar year of diagnosis (≤2005) was of no significance, leaving room to consider the non-inferiority of observation and doubt the superiority of immunotherapy for early-stage PBMZL. If corroborated by prospective studies, this conclusion is of great importance because patients can avoid overtreatment and benefit physically, psychologically, and economically, especially under the worldwide epidemic of COVID-19. Given the indolent course of this lymphoma and the non-superiority of conventional oncological modalities, the policy of watch and wait may be worthwhile considering in the management of early-stage PBMZL. For patients in need of systemic treatment, further research is warranted on novel targeted agents, which have shown significant efficacy and a manageable safety profile in relapsed or refractory EMZL (29, 30).

The staging of EMZL involving paired organs bilaterally remains contentious. Bilateral PBL was thought to be stage IV disease and associated with aggressive diseases and adverse outcomes in some publications (31, 32). It should be noted that most of those cases were diffuse large B-cell lymphoma. In our study, 10 cases with bilateral PBMZL were included and considered stage I or II. Laterality of lesions was related to the choice of treatment and half of the 10 patients received CT, which was unfavorable to survival in our results. Under these circumstances, proper stage and treatment are particularly important for bilateral PBMZL. In our study, laterality was not a predictor for OS or DSS in the univariate analysis. It seems that in the group of patients with disseminated disease, bone marrow infiltration or nodal disease rather than involvement of multiple mucosal sites is associated with a worse prognosis (24). Furthermore, 18F-FDG PET/CT finds active disease involving only the breasts in a case of PBMZL (33), supporting that bilateral involvement is a biologic phenomenon. Based on these observations, it appears to be appropriate to classify bilateral PBMZL without bone marrow or extensive nodal involvement as an early-stage disease, thus avoiding unnecessary CT.

Limitations of our study include those inherent in observational population-based studies. First, the role that other covariates describing the disease aggressiveness and individual situation, such as tumor size, laboratory indexes, and performance status, play in prognosis remains unknown on account of unavailable data from SEER. In this instance, the conclusion that CT is unfavorable to disease-specific survival has limitations to some extent as a finite number of variables were included in the multivariate analysis. The frequency and pattern of relapse, which is commonly seen in EMZL (34), is also not currently available in this database. Second, we cannot classify and assess the CT program in detail as a result of the unspecified composition of different schemes. Additionally, the group receiving no RT, Sx, or CT is not homogenous and it is unclear whether these patients underwent watch and wait strategies or immunotherapy, or other treatments. Thus, it would be better to extrapolate our conclusions to the whole population more conservatively.

## Conclusion

In conclusion, our study reviewed the clinical features, treatments, and outcomes of 370 early-stage PBMZL from the SEER database. The survival data shows the indolent course of the disease, comparable to other EMZL. Neither RT nor Sx can improve OS and DSS. Though older age, concomitant tumor, and CT predict poorer OS, only CT accounts for higher PBMZL-specific mortality. Since traditional oncological treatments fail to bring survival benefits, observation may be advisable in the management of early-stage PBMZL, and further research on novel targeted agents is warranted for patients in need.

## Data Availability Statement

Publicly available datasets were analyzed in this study. This data can be found here: https://seer.cancer.gov/data/access.html.

## Author Contributions

HL, JZ, and LQ: Conceptualization, methodology, data curation, formal analysis, and writing—original draft. LC, YM, XZ, and HS: Conceptualization, formal analysis, writing—review, and editing. LW and WX: Supervision, methodology, writing—review, and editing. JL and LF: Conceptualization, methodology, funding acquisition, supervision, writing—review, and editing. All authors contributed to the article and approved the submitted version.

## Funding

This study was supported by grants from the National Natural Science Foundation of China (81720108002), the National Science and Technology Major Project (2018ZX09734-007), and the Excellent Youth Foundation Project of Jiangsu Province (BK20160099). Translational Research Grant of NCRCH (2020ZKZB01), CSCO Research Foundation (Y-Roche2019/2-0090).

## Conflict of Interest

The authors declare that the research was conducted in the absence of any commercial or financial relationships that could be construed as a potential conflict of interest.

## Supplementary Material

Supplementary Figure 1 Kaplan-Meier survival curves stratified by treatments in the young and old patients. **(A, B)** Kaplan-Meier curves of overall survival **(A)** and disease-specific survival **(B)** stratified by treatments in the young (age≤60 years) patients. **(C, D)** Kaplan-Meier curves of overall survival **(C)** and disease-specific survival **(D)** stratified by treatments in the old (age>60 years) patients. CT, chemotherapy; RT, radiotherapy; Sx, surgery.
