# Supplementary material for: Conventional Treatments Cannot Improve Outcomes of Early-Stage Primary Breast Marginal Zone Lymphoma
Source: Front Oncol. 2021 Apr 13;10:609512. doi: 10.3389/fonc.2020.609512 (PMC8076799; doi:10.3389/fonc.2020.609512)
Supplement: Supplementary file 2 [file Table_1.docx]

Supplementary Material

**Supplementary Table 1.** Clinical characteristics of primary breast marginal zone lymphoma patients treated with or without RT before and after propensity matching

|  |  |  |  | Overall Survival | | | Disease-specific Survival | | |
| --- | --- | --- | --- | --- | --- | --- | --- | --- | --- |
| Variables | Unmatched dataset | | | Propensity score-matched (1:1) dataset | | | Propensity score-matched (1:1) dataset | | |
|  | with RT | without RT | P | with RT | without RT | P | with RT | without RT | P |
|  | (n=163) | (n=207) | value | (n=140) | (n=140) | value | (n=122) | (n=122) | value |
| Age (median, range) | 65 (24-93) | 70 (25-92) | **<0.001** | 67 (33-93) | 68 (36-90) | 0.899 | 69 (25-93) | 67 (25-92) | 0.621 |
| Sex (female) | 154 (94.5) | 201 (97.1) | 0.204 | 133 (95.0) | 134 (95.7) | 0.776 | 116 (95.1) | 116 (95.1) | 1.000 |
| Race (white) | 129 (79.1) | 185 (89.4) | **0.006** | 119 (85.0) | 123 (87.9) | 0.485 | 113 (92.6) | 108 (88.5) | 0.273 |
| Laterality (unilateral) | 161 (98.8) | 199 (96.1) | 0.196 | 138 (98.6) | 138 (98.6) | 1.000 | 120 (98.4) | 121 (99.2) | 1.000 |
| Ann Arbor stage (I) | 146 (89.6) | 177 (85.5) | 0.244 | 125 (89.3) | 126 (90.0) | 0.845 | 109 (89.3) | 110 (90.2) | 0.833 |
| Concomitant tumor (yes) | 50 (30.7) | 83 (40.1) | 0.061 | 45 (32.1) | 52 (37.1) | 0.379 | 44 (36.1) | 44 (36.1) | 1.000 |
| Calendar year of Diagnosis  (median, range) | 2009 (1998-2015) | 2008 (1998-2015) | 0.579 | 2009 (1998-2015) | 2008 (1998-2015) | 0.669 | 2008.5 (1998-2015) | 2007 (1998-2015) | 0.584 |
| Sx (yes) | 65 (39.9) | 88 (42.5) | 0.609 | 57 (40.7) | 57 (40.7) | 1.000 | 51 (41.8) | 57 (46.7) | 0.439 |
| CT (yes) | 21 (12.9) | 42 (20.3) | 0.060 | 21 (15.0) | 25 (17.9) | 0.519 | 16 (13.1) | 18 (14.8) | 0.712 |

CT, chemotherapy; RT, radiotherapy; Sx, surgery
