# Supplementary material for: Conventional Treatments Cannot Improve Outcomes of Early-Stage Primary Breast Marginal Zone Lymphoma
Source: Front Oncol. 2021 Apr 13;10:609512. doi: 10.3389/fonc.2020.609512 (PMC8076799; doi:10.3389/fonc.2020.609512)
Supplement: Supplementary file 3 [file Table_2.docx]

Supplementary Material

**Supplementary Table 2.** Clinical characteristics of primary breast marginal zone lymphoma patients treated with or without Sx before and after propensity matching

|  |  |  |  | Overall Survival | | | Disease-specific Survival | | |
| --- | --- | --- | --- | --- | --- | --- | --- | --- | --- |
| Variables | Unmatched dataset | | | Propensity score-matched (1:1) dataset | | | Propensity score-matched (1:1) dataset | | |
|  | with Sx | without Sx | P | with Sx | without Sx | P | with Sx | without Sx | P |
|  | (n=153) | (n=217) | value | (n=141) | (n=141) | value | (n=133) | (n=133) | value |
| Age (median, range) | 67 (24-91) | 69 (33-93) | 0.138 | 66 (24-91) | 68 (33-93) | 0.377 | 69 (24-91) | 68 (33-93) | 0.729 |
| Sex (female) | 145 (94.8) | 210 (96.8) | 0.336 | 135 (95.7) | 135 (95.7) | 1.000 | 128 (96.2) | 126 (94.7) | 0.555 |
| Race (white) | 132 (86.3) | 182 (83.9) | 0.525 | 123 (87.2) | 119 (84.4) | 0.495 | 115 (86.5) | 115 (86.5) | 1.000 |
| Laterality (unilateral) | 151 (98.7) | 209 (96.3) | 0.206 | 140 (99.3) | 140 (99.3) | 1.000 | 131 (98.5) | 131 (98.5) | 1.000 |
| Ann Arbor stage (I) | 138 (90.2) | 185 (85.3) | 0.160 | 127 (90.1) | 125 (88.7) | 0.699 | 118 (88.7) | 120 (90.2) | 0.689 |
| Concomitant tumor (yes) | 55 (35.9) | 78 (35.9) | 1.000 | 51 (36.2) | 46 (32.6) | 0.531 | 47 (35.3) | 49 (36.8) | 0.798 |
| Calendar year of diagnosis  (median, range) | 2006 (1998-2015) | 2010 (1998-2015) | **<0.001** | 2007 (1998-2015) | 2008 (1998-2015) | 0.803 | 2007 (1998-2015) | 2008 (1998-2015) | 0.549 |
| RT (yes) | 65 (42.5) | 98 (45.2) | 0.609 | 60 (42.6) | 61 (43.3) | 0.904 | 55 (41.4) | 59 (44.4) | 0.620 |
| CT (yes) | 26 (17.0) | 37 (17.1) | 0.988 | 24 (17.0) | 24 (17.0) | 1.000 | 23 (17.3) | 23 (17.3) | 1.000 |

CT, chemotherapy; RT, radiotherapy; Sx, surgery
